# Charge-Converting Nanoemulsions as Promising Retinal Drug and Gene Delivery Systems

**DOI:** 10.1021/acsami.2c11649

**Published:** 2022-09-20

**Authors:** Nguyet-Minh Nguyen Le, Sarah Zsák, Bao Le-Vinh, Julian David Friedl, Gergely Kali, Patrick Knoll, Hartwig Wolfram Seitter, Alexandra Koschak, Andreas Bernkop-Schnürch

**Affiliations:** † Center for Chemistry and Biomedicine, Department of Pharmaceutical Technology, Institute of Pharmacy, University of Innsbruck, Innrain 80/82, 6020 Innsbruck, Austria; ‡ Center for Chemistry and Biomedicine, Department of Pharmacology and Toxicology, Institute of Pharmacy, University of Innsbruck, Innrain 80/82, 6020 Innsbruck, Austria

**Keywords:** polyphosphate coating, charge-converting system, cell-penetrating peptide, lipid-based nanocarriers, retinal drug delivery systems, gene transfection

## Abstract

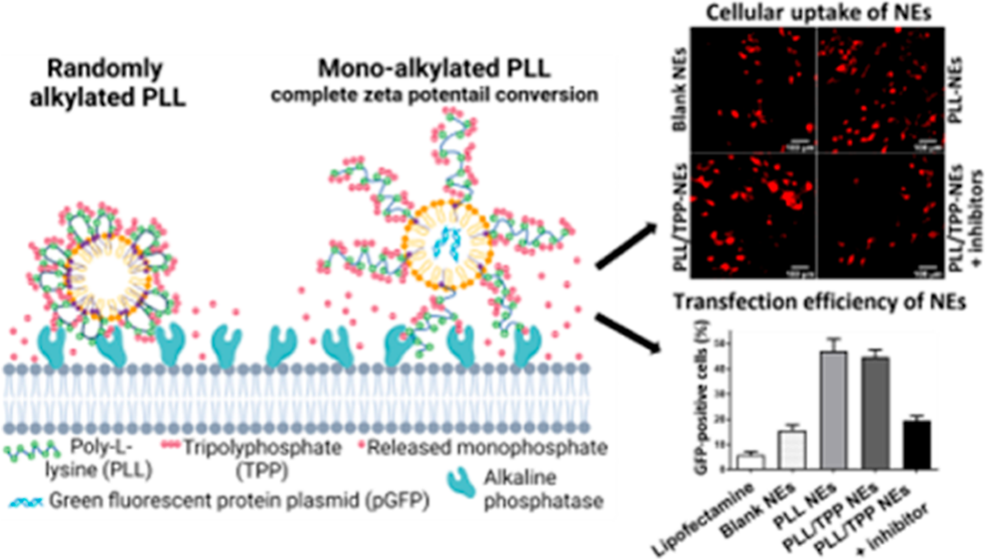

Aim: This study aimed to develop phosphatase-responsive ζ potential converting nanocarriers utilizing polyphosphate-coated cell-penetrating peptide (CPP)-decorated nanoemulsions (NEs) as a novel gene delivery system to retinal cells. Methods: Poly-l-lysine (PLL) was first conjugated with oleylamine (OA) only at its carboxylic end to form the amphiphilic PLL–oleylamine (PLOA) conjugate. Afterward, NEs were loaded with PLOA prior to being coated with tripolyphosphate (TPP) to generate PLOA/TPP NEs. A plasmid containing a reporter gene for green fluorescent protein plasmid (pGFP) was complexed with cationic surfactants forming hydrophobic ion pairs that were loaded in the oily core of NEs. Phosphate removal, ζ potential conversion, and cytotoxicity of the system were evaluated. Cellular uptake and transfection efficiency were investigated in 661W photoreceptor-like cells via microscopic analysis, fluorescence spectroscopy, and flow cytometry. Results: Dephosphorylation of PLOA/TPP NEs triggered by alkaline phosphatase (ALP) resulted in the exposure of positive amine groups on the surface of NE droplets and a notable conversion of the ζ potential from −22.4 to +8.5 mV. Cellular uptake of PLOA/TPP NEs performed on 661W photoreceptor-like cells showed a 3-fold increase compared to control NEs. Furthermore, PLOA/TPP NEs also showed low cytotoxicity and high transfection efficacy with ∼50% of cells transfected. Conclusions: Polyphosphate-coated CPP-decorated NEs triggered by ALP could be a promising nanosystem to efficiently deliver drugs and genetic materials to photoreceptor-like cells and other retinal cells for potential treatments of retinal diseases.

## 1 Introduction

Nonviral gene delivery nanosystems are promising strategies to cure and treat retinal- and photoreceptor-related diseases such as diabetic retinopathy, age-related macular degeneration, and retinal neovascularization disorders that are shown to have great potential in restoring vision loss, resolving difficulties in discerning colors, and improving light adaptation.^1−4^ However, due to the unique anatomy of the eyes, efficient gene delivery to the retina remains challenging.^5−7^ Clinically, intravitreal injection is currently the most favorable route to deliver drugs and drug/gene nanocarriers to the posterior segment of the eye as it can bypass the cornea, provide high local drug concentrations, and minimize systemic side effects.^1,8^ In spite of this fact, the insufficient transportation to the retina and gene expression of nonviral gene delivery systems are still among the biggest obstacles.^9−12^

Positively charged nanoparticles have been shown to electrostatically interact with negatively charged glycosaminoglycan and hyaluronic acid in the vitreous humor, hindering particle diffusion, promoting aggregation in some cases, and limiting particles approaching the retina.^13−15^ Besides, nanoparticle positive charges may facilitate the formation of the protein corona that could reduce nanoparticle cellular uptake.^16^ Nevertheless, electrostatic interactions between positively charged nanoparticles and negatively charged domains on cell surfaces are needed for nanoparticle uptake, which is essential for gene delivery systems.^17,18^ Therefore, a system that could provide the right surface charge at the right stage would minimize the hurdles and maximize the benefits for optimal drug delivery.

Our research group has been employing the alkaline phosphatase (ALP)-triggered charge-converting strategy that was shown to assist negatively charged phosphate-decorated nanocarriers to efficiently penetrate highly negatively charged mucus gel layers and be internalized into underneath cell layers.^19,20^ Enhancement in cellular internalization of phosphate-decorated nanocarriers was caused by the positive charges formed on the surface of the nanocarriers as they approached the cell and were cleaved off their phosphate groups by membrane-bound ALP. As ALP is shown to be present in retinal tissue, that is, the inner and outer segments of the cone photoreceptors, the inner and outer plexiform layer of cone axons, and Müller cells,^21−24^ ALP-triggered charge-converting nanosystems that could avoid interactions with negatively charged proteins in the vitreous humor and be efficiently internalized into retinal cells are of interest. These charge-converting nanocarriers which can be introduced to the eye by intravitreal injection or transscleral delivery systems using microneedles^25,26^ could be a potential approach to improve gene and drug delivery efficiencies to the retina.

In this study, we designed ALP-responsive polyphosphate-coated cell-penetrating peptide (CPP)-decorated nanoemulsions (NEs) aiming at the delivery of drug or plasmid DNA to the retina and photoreceptor cells. NEs have been shown to be attractive and efficient nonviral gene delivery systems as they can provide high gene loading capacity, protection of cargo genes from enzymatic degradation, high transfection efficacy, and low toxicity.^27−29^ We prepared NEs by diluting lipophilic preconcentrates loaded with CPP, drug, or genetic materials. First, poly-l-lysine (PLL), a CPP, was grafted with a fatty acid chain so that it could anchor itself on the exterior of NEs. The highly positively charged surface of the obtained NEs was subsequently coated with tripolyphosphate (TPP) via electrostatic interactions. Finally, TPP-coated PLL-decorated NEs loaded with Lumogen red or a plasmid encoding green fluorescent protein plasmid (pGFP) were evaluated for cellular uptake enhancement and transfection efficiency in 661W photoreceptor-like cells.

## 2 Materials and Methods

### 2.1 Materials

Poly-l-lysine hydrobromide (PLL; 4.2 KDa) was purchased from Alamanda Polymers (Alabama, USA). Dulbecco’s modified Eagle’s medium (DMEM) Opti-MEM, RIPA lysis, and extraction buffer were obtained from Thermo Scientific (Vienna, Austria). ALP from bovine intestinal mucosa ≥10.0 mg protein/mL (≥6500 DEA units/mg protein), phosphatase inhibitors cocktail 2 (PIC2), Triton-X-100, and Trypan blue 0.4% solution for molecular biology, di*tert*-butyl dicarbonate 99% (Boc), trifluoroacetic acid 99% (TFA), 2, 4-(2-hydroxyethyl)-1-piper-azineethanesulfonic acid ≥99.5% (titration) (HEPES), potassium phosphate monobasic 99.5–100.5% (KH_2_PO_4_), sodium acetate anhydrous ≥99%, glacial acetic acid ≥99%, ammonium molybdate tetrahydrate ≥99.0% (titration), sodium dodecyl sulfate ≥99.0% (SDS), ascorbic acid ≥98% (with iodine, titration), 2-(*N*-morpholino)ethanesulfonic acid hydrate ≥99% (titration) (MES), 2,4,6–trinitrobenzenesulfonic acid (TNBS), l-cysteine hydrochloride ≥98%, isopropyl myristate ≥98%, polysorbate 80 (HLB = 15), sodium tripolyphosphate ≥98.0% (titration), hexadecyltrimethylammonium bromide (CTAB) ≥ 99%, and 1,2-dioleoyl-3-trimethylammonium-propane chloride (DOTAP) ≥ 98% were purchased from Sigma-Aldrich (Vienna, Austria). Capmul MCM (caprylic/capric mono- and diglycerides, HLB = 6.7) was kindly provided by Abitec (Oberhausen, Germany). Lumogen F red was obtained from Kremerpigmente GmbH & Co., KG (Aichstetten, Germany). pGFP was a gift from the Department of Pharmacology and Toxicology, University of Innsbruck, Austria. All chemicals were of analytical grade and purchased from commercial sources.

### 2.2 Methods

#### 2.2.1 Synthesis of the PLL–Oleylamine Conjugate

**Figure 1:**
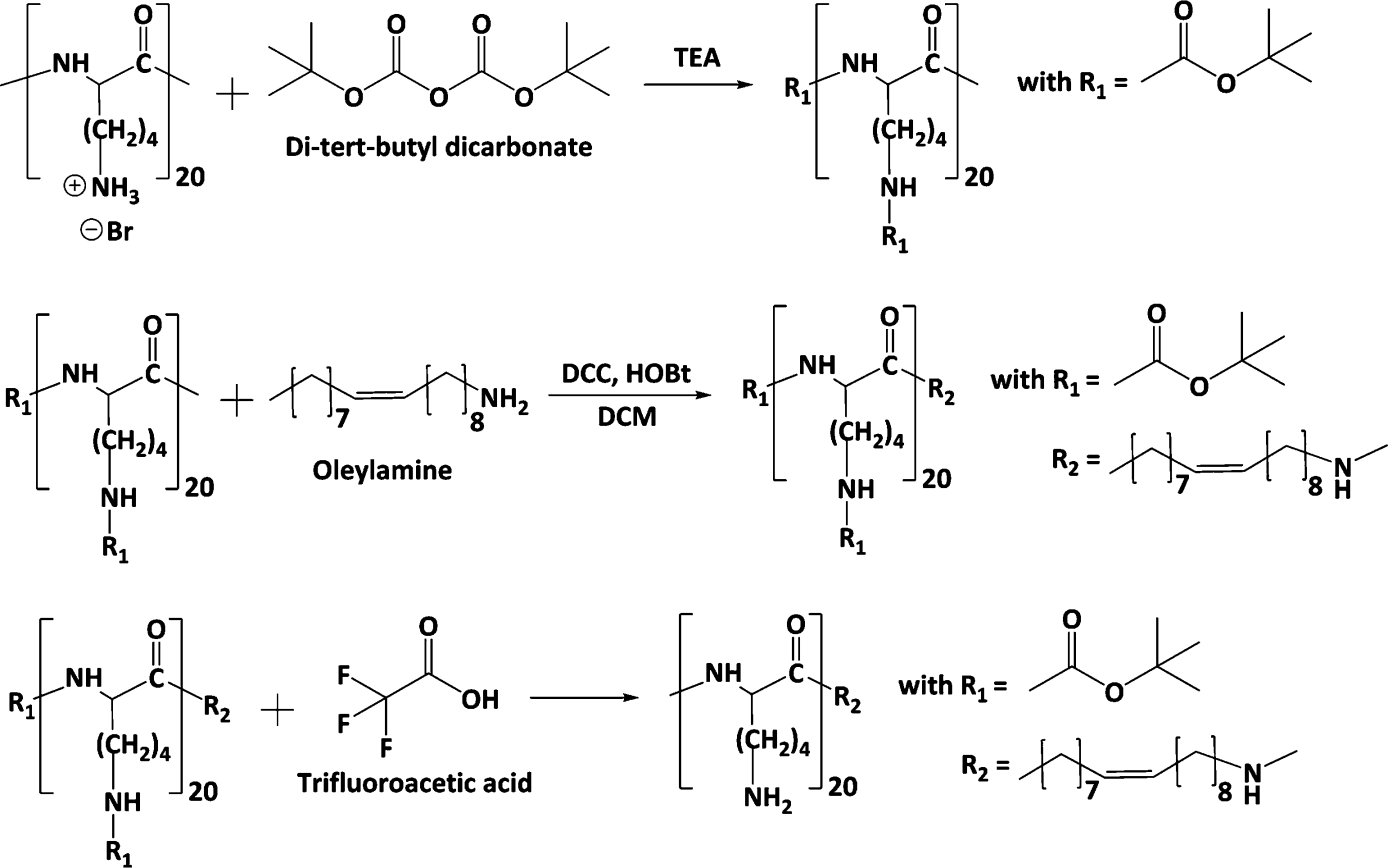
Synthesis of PLOA. PLL was 100% Boc-protected prior to the alkylation with oleylamine. The attachment of oleylamine to PLL occurred at the carboxylic end of PLL.

As illustrated in Figure 1, oleylamine was anchored at the carboxylic end of the PLL backbone via the amidation reaction to yield the PLL–oleylamine conjugate (PLOA). In brief, 100 mg of PLL (0.47 mmol of primary amine groups) were dissolved in 3 mL of anhydrous methanol containing 0.71 mmol trimethylamine. Subsequently, 0.71 mmol Boc dissolved in 2 mL of methanol was slowly added dropwise into the PLL solution. After 2 h of stirring at room temperature, the mixture was monitored by thin-layer chromatography (TLC) to ensure completion of the Boc protection reaction. Methanol was removed from the mixture using a rotary evaporator. The obtained solid substances were solubilized in dichloromethane (DCM). DCC (0.94 mmol) and HOBt (0.94 mmol) were added to activate the carboxylic group on Boc-protected PLL (Boc-PLL). The mixture was kept in an ice bath and stirred for 30 min. The activated Boc-PLL was then added dropwise to an oleylamine (0.94 mmol) solution in DCM being stirred at room temperature. The amidation reaction was controlled using TLC. The reaction mixture was subsequently washed using a 0.1 M NaCl solution (10 mL, 3 times) and deionized water (DIW) (10 mL, 3 times). The organic phase was collected and evaporated. The obtained solid was stirred with 6 mL of a 95% (v/v) TFA solution for 4 h to remove Boc groups. After the deprotection reaction, the reaction mixture was added with 10 mL of DIW, adjusted to pH 7, and extracted with DCM (15 mL, 5 times). The aqueous phase was concentrated using a rotary evaporator and purified using a Sephadex G-10 column. The collected fractions were monitored using TLC. PLOA-containing fractions were pooled and subsequently freeze-dried (Gamma LSC 1-16, Martin Christ, Germany). The PLOA powder was kept at −20 °C for further use.

The structure of PLOA was further confirmed by ^1^H NMR. Measurements were performed on a “Mars” 400 MHz AVANCE 4 Neo spectrometer from Bruker Corporation (Billerica, MA, USA, 400 MHz) in dimethyl sulfoxide-d_6_ (DMSO-d_6_) solution at 24 °C. Chemical shifts are expressed in parts per million (ppm).

#### 2.2.2 Lipophilic Preconcentrates and NE Preparation and Characterization

The lipophilic preconcentrate is composed of isopropyl myristate, Capmul MCM, polysorbate 80, and propylene glycol as the oil, cosurfactant, surfactant, and cosolvent, respectively. The final ratio of 35:40:15:10 (w/w) was used for further experiments. The blank lipophilic preconcentrate was subsequently loaded with PLOA or oleylamine as controls. The payload of PLOA in the lipophilic preconcentrate was 0.3% w/w. Lipophilic mixtures were shaken in a thermomixer set at 1800 rpm and 40 °C for 24 h (ThermoMixer C, Eppendorf, Germany).

The lipophilic preconcentrate of each NE formulation was stored at room temperature for 3, 6, and 9 months for long-term stability testing. At the predetermined time points, samples were centrifuged at 13,400 rpm for 15 min and evaluated for possible phase separation.

The NE was prepared by diluting the lipophilic preconcentrate in DIW. Emulsification was carried out by gently pipetting the mixture 2–3 times or gently and briefly vortexing the mixture for 2–3 s. NEs at concentrations of 0.2% (v/v) were tested for the droplet size, ζ potential, and polydispersity index (PDI) via photon correlation spectroscopy using a Zetasizer Nano-ZSP instrument (Malvern Instruments, Worcestershire, UK). The droplet size and PDI were determined in backscatter (173°) detection mode. ζ potential measurement was carried out using a Dip cell (Malvern ZEN 1002). All measurements were executed at 37 °C at least three times.

#### 2.2.3 Anionic Coating of NE Droplets with Tripolyphosphate

The PLOA-loaded lipophilic preconcentrate was diluted five times in DIW to obtain PLOA-decorated NEs (PLOA NEs). To obtain anionic coating layers on the surface of the emulsion droplets, 250 μL of 14% (w/v) of TPP solution was added dropwise slowly to the diluted PLOA NE with gentle vortexing. Afterward, all mixtures were mixed for 30 min in a thermomixer set at 300 rpm and 24 °C. Unbound polyphosphates were removed using a Vivaspin ultrafiltration device (membrane cutoff: 10 kDa). Concentrated NEs were then diluted with 2 mL of DIW and washed. At least three wash cycles were done until a constant ζ potential was reached. The final concentrated TPP-coated PLOA-decorated NEs (TPP/PLOA NEs) were diluted in HEPES-buffered saline (HBS) to a final concentration of 1%, 0.05%, or 0.005% (v/v) for further experiments. HBS is composed of 1 g/L dextrose, 20 mM HEPES, 5 mM KCl, 136.7 mM NaCl, and 1 mM CaCl_2_ adjusted to pH 6.8 using 1 M NaOH.

#### 2.2.4 Enzymatic Monophosphate Cleavage and ζ Potential Conversion by ALP

ALP is shown to quickly dephosphorylate not only TPP but also a variety of polyphosphates.^30−32^ In brief, two units of isolated ALP were spiked into 1 mL of 1% (v/v) NEs, and the mixture was incubated in an orbital shaker–incubator at 37 °C under gentle shaking. At time points of 5, 15, 30, 45, 60, 90, 120, and 180 min, an aliquot of 100 μL was withdrawn from each sample and compensated with 100 μL of fresh prewarmed HBS. Subsequently, 700 μL of 10% SDS in 0.1 M sodium acetate buffer pH 4.0 (SDS-buffer pH 4.0) was added to terminate the enzymatic cleavage reaction. To quantify released monophosphate, 100 μL of 1% (w/v) ammonium molybdate solution and 100 μL of 1% (w/v) ascorbic acid were added. The mixture was incubated at 37 °C for 60 min in the orbital shaker–incubator. The absorbance of samples was measured at 870 nm using a microplate reader (Tecan Infinite M200 spectrophotometer, Tecan Austria GmbH, Grödig, Austria). A series of standard solutions with increasing amounts of K_2_HPO_4_ were utilized to generate the standard curve and calculate the released monophosphate amount. Negative control and blank were prepared in the same manner but using a buffer without isolated ALP and a buffer without NEs, respectively. Triplicate samples for each NE were performed. This assay is specific for inorganic monophosphate, and there is no color development with TPP.

The ζ potential of the PLOA/TPP NE before and after incubation with isolated ALP was measured using Zetasizer Nano-ZSP (Malvern Instruments, Worcestershire, UK). Prior to ζ potential measurement of PLOA/TPP NEs after incubation with ALP, cleaved monophosphate was removed using a centrifugal concentrator Vivaspin (10 KDa) as previously described in 2.2.3. The washing step was repeated until negligible monophosphate was found in the flow-through solution. For this experiment, three washing cycles were done. DIW was then added to the concentrated NE to recover the initial volume. All ζ potential measurements were performed at 37 °C utilizing a Dip cell device (Malvern ZEN 1002).

#### 2.2.5 Determination of Phosphatase Activity in the Pig Eye Vitreous Humor

After being injected via the vitreal pathway, anionic coating NEs must retain their surface charge unchanged to permeate through the negatively charged vitreous humor before approaching the target cells. To further confirm the vitreous will not disturb the phosphate coatings of PLOA/TPP NEs, we studied the phosphatase activity in the vitreous body isolated from pig eyes. In brief, pig eyes were freshly obtained from a slaughterhouse and the vitreous fluid was extracted using a 5 mL syringe. The vitreous fluid of four eyes was collected and centrifuged for 5 min at 5000 rpm (ThermoMixer C, Eppendorf, Germany). The phosphatase activity in the supernatant was analyzed by a colorimetric assay. Accordingly, 500 μL of vitreous fluid was mixed with 500 μL of 10 mM *p*-nitrophenyl phosphate (PNP) in a 0.5 M 2-amino-2-methyl-1-propanol (AMP) buffer, supplemented with 4.9 mM MgCl_2_ and hydrochloride acid in a final concentration of 0.5 M. PNP is a substrate of ALP and transformed by ALP to 4-nitrophenol that can be detected photometrically at 405 nm. Vitreous fluid spiked with 1 U ALP, vitreous fluid spiked with 1 mU ALP, buffer containing 1 U, and buffer containing 1 mU ALP served as controls. Samples were incubated for 3 h at 37 °C. At different time points, 50 μL aliquots were withdrawn and phosphatase activity was stopped by adding 50 μL of 1 M NaOH. A calibration curve of dilutions of a 4-nitrophenol solution was prepared for quantification.

#### 2.2.6 Cytotoxicity

661W photoreceptor-like cells were used up to cell passage number 100. Cells were cultured in DMEM supplemented with 10% fetal bovine serum, 2 mM l-glutamine, and 0.2% penicillin–streptomycin. Cells were kept in a 5% CO_2_ and 90% relative humidity atmosphere at 37 °C in a cell incubator. The 661W cells were seeded in a 24-well plate at a density of 5 × 10^3^ cells/well and cultivated for 4 days to obtain about 80% confluency. The medium was changed on the first day after seeding and every 2 days.

Cytotoxicity of PLOA and PLOA/TPP NEs was evaluated via the 3-(4, 5-dimethylthiazolyl-2)-2, 5-diphenyltetrazolium bromide (MTT) assay. A 1:10 MTT working solution in sterile HBS buffer pH 6.8 was prepared from a MTT stock solution (5 mg/mL in HBS; stored at −20 °C). Prior to the test, the cell monolayer was washed twice with prewarmed sterile HBS and incubated with different concentrations of NEs for 1 and 3 h. HBS and 1% v/v Triton-X 100 in HBS served as the negative control and positive control, respectively. After NE removal and two washing steps with prewarmed HBS, 100 μL of prewarmed MTT working solution was added to each well and incubated for 3 h. Subsequently, the MTT solution was aspirated and the resulting formazan crystals were dissolved with 100 μL of DMSO. The absorbance of the formazan solution in DMSO was recorded at 570 nm using a microplate reader (Tecan Infinite M200 spectrophotometer, Tecan Austria GmbH, Grödig, Austria). Cell viability was assessed by the metabolic capability of viable cells to reduce MTT to purple formazan crystals in comparison with positive control and negative control (eq 1)
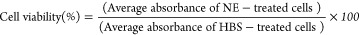
1

#### 2.2.7 Phosphate Cleavage by Cellular Enzymes

The release of monophosphate release from the NE surface into the cell culture medium was determined in the presence and absence of phosphatase inhibitors. Prior to the experiment, 661W cells were washed twice with prewarmed HBS and incubated with 500 μL of HBS with or without 0.5% v/v PIC2 in HBS buffer for 30 min. Thereafter, cells were incubated with 250 μL of 0.05% v/v of PLOA/TPP NEs solved in prewarmed HBS for the times indicated, that is, 0, 30, 60, 90, 120, 150, and 180 min. HBS alone served as the control. An aliquot of 70 μL was taken from each well, supplemented with 70 μL of SDS-buffer pH 4.0 and 10 μL of 1% w/v ammonium molybdate solution followed by 10 μL of 1% (w/v) ascorbic acid. The mixture was incubated at 37 °C for 60 min prior to the absorbance measurement at 870 nm. A serial dilution of K_2_HPO_4_ was used as the standard curve for quantifying free monophosphate. After sampling, the test emulsion was removed from each well and the cell layer was gently washed twice with prewarmed phosphate buffer saline (PBS). 500 μL of PBS and 150 μL of radioimmunoprecipitation assay (RIPA) buffer were subsequently added to initiate cell lysis. The protein content was quantified via a Pierce Micro BCA protein assay kit following the supplier’s instructions. The amount of released monophosphate was calculated from triplicate samples at every time point for each NE (eq 2).
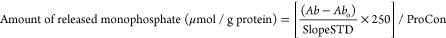
2where Ab: absorbance of NE-treated cells at 870 nm, Ab_o_: absorbance of HBS-treated cells at 870 nm, SlopeSTD: slope value of K_2_HPO_4_ standard curve, and ProCon: protein content in cell lysate.

#### 2.2.8 Cellular Uptake Studies

Concentration-dependent cellular uptake of NEs was screened by fluorescence spectroscopy. Microscopic analysis was used to visualize the cellular uptake of NEs at a concentration of 0.005% v/v. Further examination of cellular uptake efficiency was performed by flow cytometry using a NE concentration of 0.05% v/v.

For the cellular uptake experiment, Lumogen red was loaded into the lipophilic phases of control NEs, PLOA NEs, and PLOA/TPP NE formulations at 0.1% w/w. 661W cells were cultured for 4 days in 24-well plates as described in 2.2.6. After washing and equilibrating with HBS for 30 min, cell layers were incubated with 250 μL of different concentrations of NEs for 60 min. In order to evaluate the effect of the surface charge role of ALP on cellular uptake of NEs, 661W cells were treated with 0.5% v/v PIC2 in HBS prior to and during the incubation with NEs. Subsequently, tested samples were aspirated and cell layers were gently washed twice with prewarmed PBS.

For quantification of NEs internalized in cells by fluorescence spectroscopy, cells were lysed and the amount of internalized NEs per 1 g of cell protein was calculated. Accordingly, 500 μL of PBS and 150 μL of ice-cold RIPA buffer for lysis were added to cell layers. Cell lysates were collected and the fluorescence intensity was measured at an excitation wavelength λ_ex_ = 575 nm and an emission wavelength λ_em_ = 610 nm. A standard curve was built from a serial dilution of Lumogen-loaded NEs with nontreated cell lysate. A Pierce Micro BCA protein assay kit with bovine serum albumin as the standard protein was used for protein content quantification. The amount of internalized NEs was calculated by the average uptake amount of NEs from triplicate experiments (eq 3).
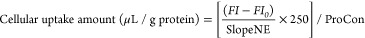
3where FI: fluorescence intensity of NE-treated cell lysate, FI_0_: fluorescence intensity of HBS-treated cell lysate, SlopeNE: slope from linear regression analysis of NE standard curve, and ProCon: protein content in each cell lysate.

The cellular uptake efficiency was observed with an inverted fluorescence microscope (Zeiss Axio Observer) using a 10× objective with a numerical aperture of 0.45. NEs at a concentration of 0.005% v/v were used in microscopic imaging experiments. After incubation with test samples, cells were washed twice with Opti-MEM and observed under a microscope. A filter at λ = 555 nm and excitation light (LED) λ = 567 nm was utilized. The same parameters were set up for all experiments for comparability of the fluorescence intensity of all formulations. Image postprocessing was performed using ImageJ software.

Furthermore, the quantification of NE cellular uptake was examined using a flow cytometer (BD FACSAria III Cell Sorter). NEs at a concentration of 0.05% v/v were used. The fluorescence intensity of diluted NEs among all formulations was checked for equality using a spectrometer (Tecan Infinite M200 spectrophotometer, Tecan Austria GmbH, Grödig, Austria) prior to the uptake study. Single cells were collected by incubation of cell monolayers with 200 μL of Accutase for 10 min. Cell suspensions pooled from six wells of the same sample were transferred to a 15 mL Falcon centrifuge tube. Cell pellets were obtained by centrifugation at 800 rpm for 5 min, and the medium was discarded and replaced with 2 mL of ice-cold PBS. The wash cycle was done twice. Cell pellets were subsequently resuspended in 500 μL of PBS and filtered through a 70 μm-pore size cell strainer for flow cytometry analysis. The same channel voltage and gating strategy (Supporting Information Figure S.1) were maintained throughout the analysis process. The gating strategy was based on the area of forward versus side scatter (FSC-A vs SSC-A). A number of 100,000 events were analyzed for fluorescence signals. The percentage of fluorescence-positive cells was identified in the final sorted population.

#### 2.2.9 Preparation of Hydrophobic Ion Pairs of Plasmid DNA

pGFP is a common plasmid DNA used to study gene transfection efficacy of gene delivery systems on different cells and tissues. Being transfected with pGFP, cells will express green fluorescence protein that can be observed and examined by fluorescence microscopy and a flow cytometer. In order to investigate the transfection efficiency of PLOA/TPP NEs, pGFP was loaded into the lipophilic phase. To do so, hydrophobic ion pairs between pGFP and cationic surfactants were prepared to increase the lipophilicity of pGFP. In brief, pGFP was mixed with CTAB or DOTAP in a thermomixer at a molar ratio of 1:2. The resulting hydrophobic ion pairs precipitated out and were collected by centrifugation at 13,400 rpm for 5 min. The pGFP/CTAB and pGFP/DOTAP complexes were lyophilized and stored at −20 °C for further use.

#### 2.2.10 Gene Transfection Study

661W cells were cultured as described in Section 2.2.6 to reach a confluency of about 80%. The pGFP/CTAB or pGFP/DOTAP complex was loaded in the lipophilic phase at a concentration of 0.2% w/w. The pGFP complex-loaded NEs were diluted with HBS buffer to a concentration of 0.05% v/v. In order to initiate transfection studies and evaluate the effect of ALP on transfection efficiency, 250 μL of diluted NEs with or without the presence of 0.5% PIC2 was added to each well. Serving as controls, naked pGFP solution and pGFP–Lipofectamine 2000 complex (prepared according to the manufacturer’s instructions with some modifications—see the Supporting Information) in the final volume of 250 μL were also incubated with the cell layer. The ratio of pGFP/Lipofectamine 2000 in the positive control pGFP–Lipofectamine 2000 complex was 1:0.6 w/w. The final concentration of Lipofectamine 2000 on the cell layer was 0.05% v/v. The plasmid DNA amount incubated in each well was kept at ∼200 ng (per 250 μL) for all tested samples and controls. After 1 h of incubation, test samples were removed and 500 μL of fresh culture medium was added to each well. Cell plates were brought back to the cell incubator. 48 h post-transfection, cells were detached using Accutase and quantified for green fluorescent protein (GFP) expression using flow cytometry. The channel voltage and gating strategy were set in the same way as described in Section 2.2.8.

#### 2.2.11 Statistical Data Analysis

Statistical data analyses were performed using Student’s *t*-test to analyze the significant difference between two mean values. The level of *p* ≤ 0.05 was set for significant, *p* ≤ 0.01 for very significant, and *p* ≤ 0.001 for highly significant. The obtained results were expressed as the mean of at least three experiments ±standard deviation (SD).

## 3 Results and Discussion

### 3.1 Characterization of PLOA

**Figure 2:**
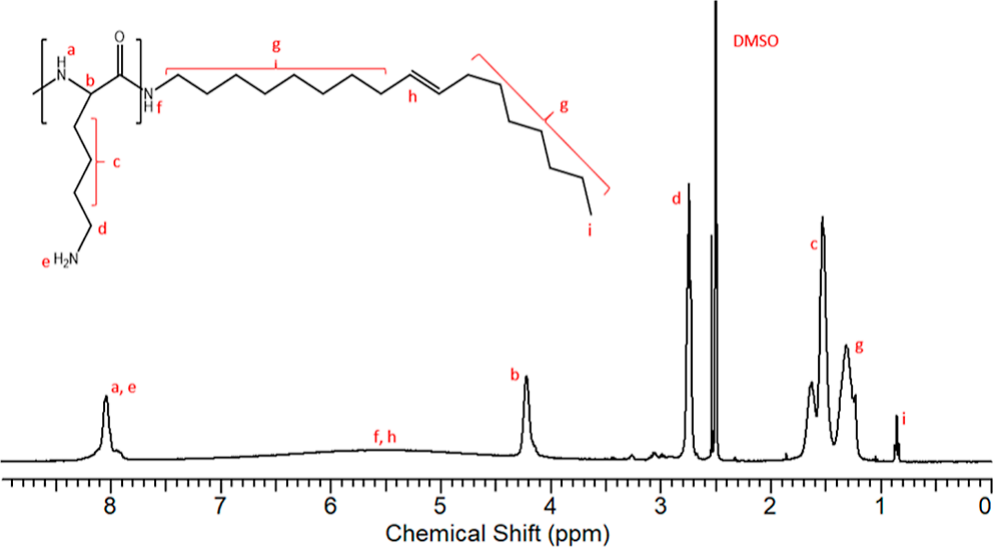
^1^H NMR spectra of PLOA in DMSO-d_6_.

The structure of PLOA was studied and confirmed by ^1^H NMR spectroscopy in DMSO-d_6_ (Figure 2). There are characteristic peaks for the PLL structure between 7.80 and 8.20 ppm associated with primary and secondary amines. The distinct peak at 4.20 ppm is associated with the methine proton, while the peaks at 1.50–1.70 and 2.80 ppm correspond to the methylene units in the lysine side chain and to the methylene proton next to primary amine in the lysine side chain. The oleyl chain was characterized by chemical shifts at 1.20–1.40 and 0.85 ppm regions corresponding to methyl and methylene protons of the hydrophobic part. A broad chemical shift from 4.50 to 7.00 ppm is due to the hydrogen bonding of amide, overlapping with the methine of the bound hydrophobic oleyl part. From the integral values of the methine of PLL and the methylene chain end of the oleyl group, the degree of modification is around 80%.

### 3.2 Preparation and Characterization of TPP-Coated NEs

As presented in Table 1, loading fatty amine into a lipophilic phase of the blank formulation led to an increase in the droplet size (*p* < 0.05, Student’s t-test), while there was no significant difference in PDI (*p* > 0.05, Student’s *t*-test). The size and PDI of PLOA NEs increased significantly, ∼1.5- and ∼1.9-fold higher compared to those of the blank formulation, respectively (*p* < 0.05, Student’s *t*-test). There was no significant difference in the size of control NEs and PLOA NEs (*p* > 0.05, Student’s *t*-test). Being coated with polyphosphates, the droplet size of PLOA NEs increased significantly, ∼1.3-fold higher than that of the PLOA NEs (*p* < 0.05, Student’s *t*-test). Regarding the ζ potential, the loading of OA into a blank formulation led to a less negative value, while the loading of PLOA led to a shift from a negative to a positive value, providing evidence for the residence of PLL on the surface of the oil droplets. When PLOA NEs were coated with TPP, the ζ potential reversed to a low negative value.

**Table 1:** Average Size, PDI, and ζ Potential Profiles of NEs^a^

formulation	loading content (%, w/w)	size (nm)	PDI	ζ (mV)	potential
blank NEs			72.9 ± 0.6	0.08 ± 0.01	–11.9 ± 0.4
control NEs	OA	0.3	95.1 ± 0.5	0.10 ± 0.01	–9.7 ± 0.2
PLOA NEs	PLOA	0.3	106.4 ± 0.7	0.16 ± 0.01	+10.0 ± 0.6
PLOA/TPP NEs	PLOA	0.3	146.1 ± 13.3	0.25 ± 0.02	–22.4 ± 1.7

a Data are means of triplicate measurements ±SD, *n* ≥ 3. OA and PLOA were loaded into a lipophilic phase of a blank formulation. TPP was coated on the surface of PLOA-decorated NEs (PLOA NEs), generating TPP-coated PLOA-decorated NEs (PLOA/TPP NEs).

The long-term stability of all NEs was evaluated after 3, 6, and 9 months of storage at room temperature. There was no phase separation observed after the centrifugation of the lipophilic phases. Upon being emulsified in DIW, there was also no significant change in average size, PDI, and ζ potential.

### 3.3 Enzymatic Cleavage of Monophosphate Inducing ζ Potential Conversion

**Figure 3:**
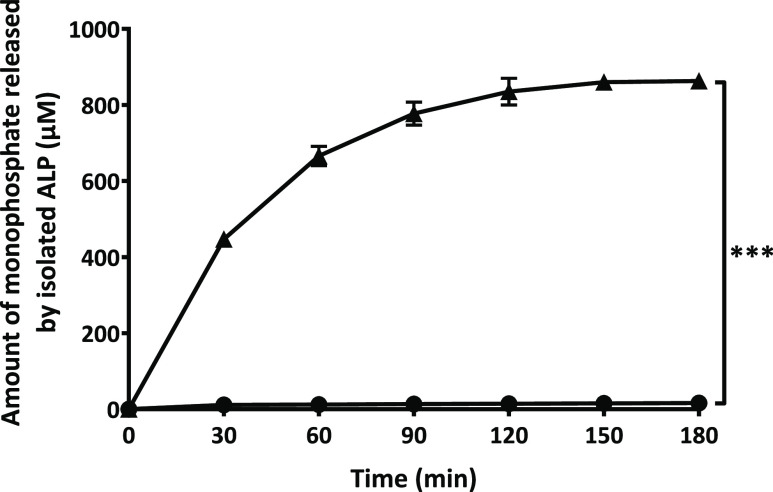
Time-dependent monophosphate release from TPP-coated PLOA-decorated NEs mediated by isolated ALP (▲) and control experiment without the addition of the enzyme (●). Data are expressed as means ± SD, *n* ≥ 3. The significant difference after 3 h is indicated as ***p < 0.001.

**Figure 4:**
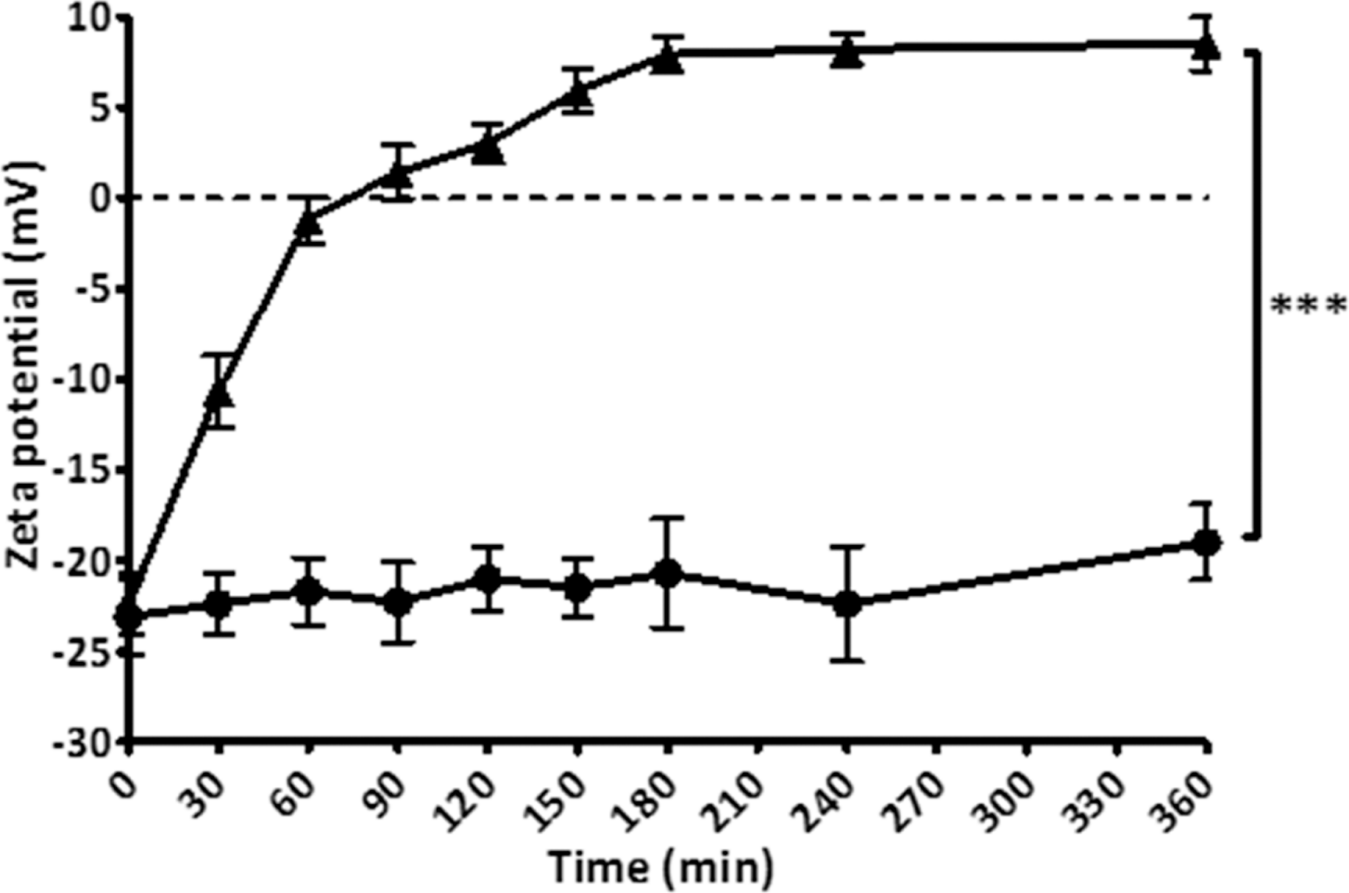
Time-dependent ζ potential conversion of TPP-coated PLOA-decorated NEs induced by isolated ALP (▲). Control experiment without the addition of the enzyme (●). Data are expressed as means ± SD, *n* ≥ 3. The significant difference is indicated as ***p < 0.001.

**Figure 5:**
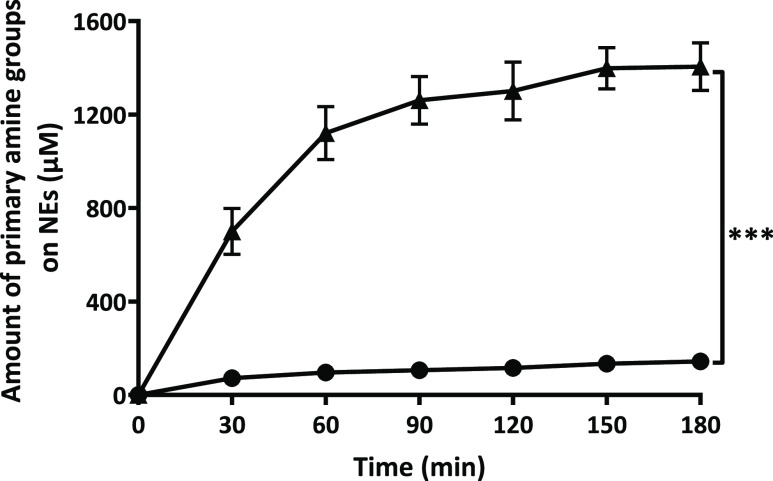
Quantification of amine moieties on the surface of TPP-coated PLOA-decorated NEs in the presence of isolated APL (▲), in the absence of ALP, served as the control (●). Data are expressed as means ± SD, *n* ≥ 3. The significant difference after 3 h is indicated as ***p < 0.001.

As illustrated in Figure 3, monophosphate release from PLOA/TPP NEs increased rapidly within the first hour and was likely to reach a plateau after 2.5 h. Without the addition of phosphatase, almost no monophosphate was detected. Together with the enzymatic cleavage of monophosphates, the ζ potential of PLOA/TPP NEs reversed from −22.4 to +8.5 mV at 3 h (Figure 4). No significant change in the ζ potential was observed for PLOA/TPP NEs without enzyme addition. In order to confirm the removal of TPP and the subsequent exposure of amine moieties on the surface of oily droplets, the TNBS assay was exploited to quantify the amount of surface-exposed primary amine groups as previously described in ref (32). The results showed an increase in the amount of surface-exposed primary amine in accordance with the increase in monophosphate release (Figure 5), confirming the gradual disappearance of the TPP coating layer and reappearance of PLL substructures on the NE surface.

**Figure 6:**
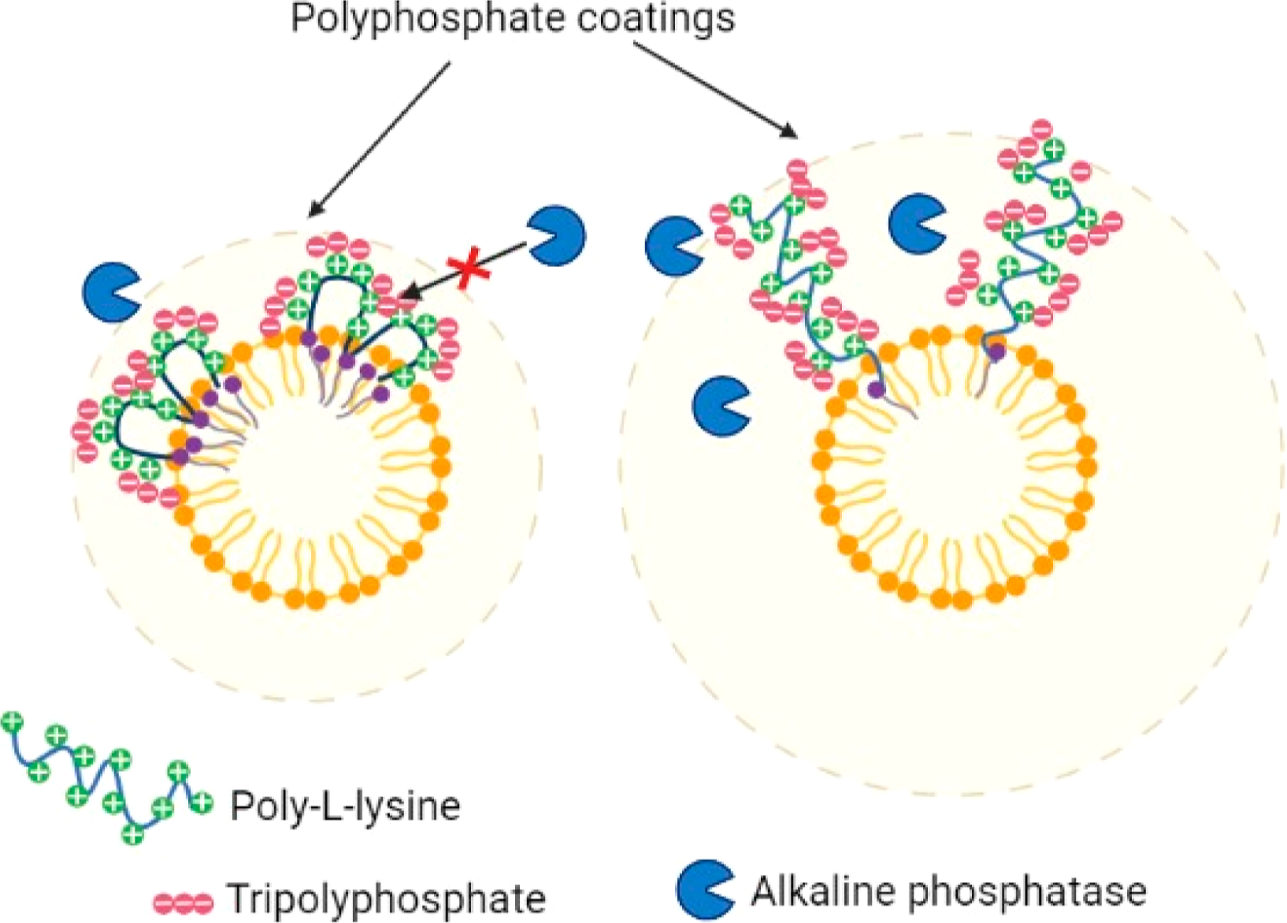
Proposed mechanism explaining the better efficacy of monoalkylated PLL (at the carboxylic end) over randomly alkylated PLL (at amine groups) on the formation of tripolyphosphate coating layer and ζ potential conversion by isolated ALP.

In this research, a PLOA conjugate as a monoalkylated PLL was synthesized. The ζ potential of PLOA/TPP NEs was converted from a low negative value of −22.4 to +8.5 mV which is nearly equal to the ζ potential value of PLOA NEs, that is, +10 mV. In a recent research study, PLL was randomly alkylated at several amine groups distributed along the polymer backbone with stearoyl chloride (SA), resulting in a higher lipophilic PL-SA conjugate. However, the ζ potential value of TPP-coated PL-SA-decorated NEs (PL-SA/TPP NEs) was not high and cannot be converted to the value of PL-SA-decorated NEs (PL-SA NEs); that is, the ζ potential of PL-SA/TPP NEs was −14 mV and was converted to +4.2 mV triggered by ALP, whereas the ζ potential of PL-SA NEs was +11 mV.^32^ We supposed that the significantly lower ζ potential value of PLOA/TPP NEs in this research (−22.4 mV vs −14 mV of PL-SA/TPP NEs) was due to the better extension of positively charged PLL heads toward the aqueous phase, facilitating the formation of the TPP coating layer by electrostatic interactions, whereas PL-SA was kept firmly on the PL-SA NEs surface, leading to poorer interactions with TPP (Figure 6). This difference in the posture of PLL heads may also affect the enzymatic cleavage since some TPP molecules may also localize close to the PL-SA/TPP NE surface, making it harder for the enzyme to access and hydrolyze. Eventually, the ζ potential conversion is incomplete.

**Figure 7:**
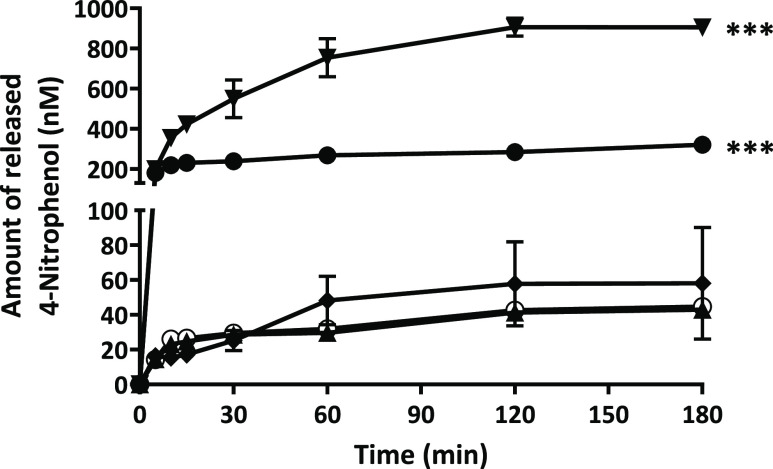
Time-dependent 4-nitrophenol release after incubation of 0.5 mL of vitreous fluid (◯), vitreous fluid with the addition of 1 U ALP (●) and 1 mU ALP (▲), and AMP-buffer incubated with 1 U (▼) and 1 mU ALP (◆) with 10 mM PNP. The significant difference after 3 h is indicated as ***p < 0.001 compared with all other samples.

**Figure 8:**
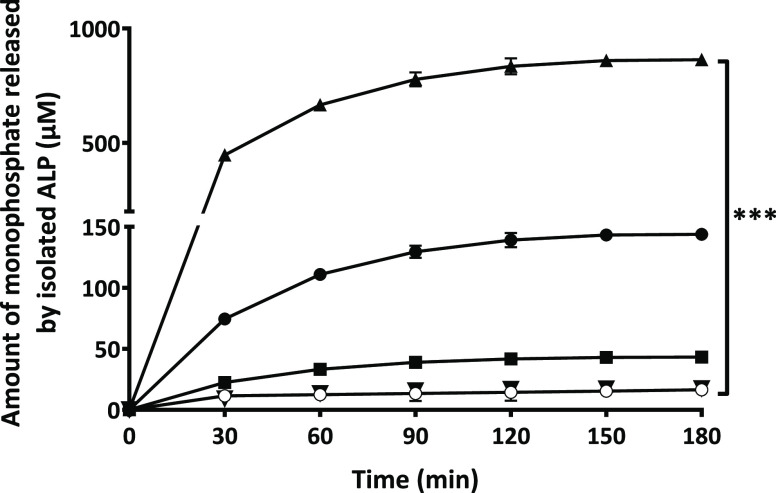
Time-dependent monophosphate release profile of TPP-coated PLOA-decorated NEs (PLOA/TPP NEs) incubated with 2 U (▲), 1 U (●), 0.5 U (■), 0.3 U (▼) of isolated ALP. The control experiment was the one without the addition of enzyme (◯). Data are expressed as means ± SD, *n* ≥ 3. The significant difference is indicated as ***p < 0.001.

After being injected via the vitreal pathway, anionic coating PLOA/TPP NEs must remain their surface charge and structure unchanged to permeate through the negatively charged vitreous humor before approaching retina cells. Understanding the phosphatase activity in the vitreous humor helps to ensure that PLOA/TPP NEs stay unimpaired when permeating through the vitreous humor. Figure 7 shows that ALP activity may be present in the vitreous humor but at a very low level, less than 1 mU per 0.5 mL, approximately. This is in accordance with Reis et al. claiming that phosphatase activity was not found in the vitreous body.^33^ The human vitreous humor volume is 4–5 mL;^34,35^ therefore, the total activity of ALP in the eye vitreous humor, if any, is less than 10 mU, assuming the properties of human and pig eye vitreous humor are similar. Data on time-dependent monophosphate release from PLOA/TPP NEs mediated by isolated ALP at different concentrations showed that hardly any released monophosphate was detected at the ALP activity of 300 mU (Figure 8) after 3 h. Concurrently, no change in ζ potential was observed. With this supporting information, we can safely say that none or a negligible amount of phosphate can be cleaved from the surface of PLOA/TPP NEs in the vitreous body. Subsequently, there would be no ζ potential conversion triggered before NEs reach the retinal cells.

### 3.4 Monophosphate Release from Polyphosphate-Coated CPP-Decorated NEs by Cellular Enzymes

**Figure 9:**
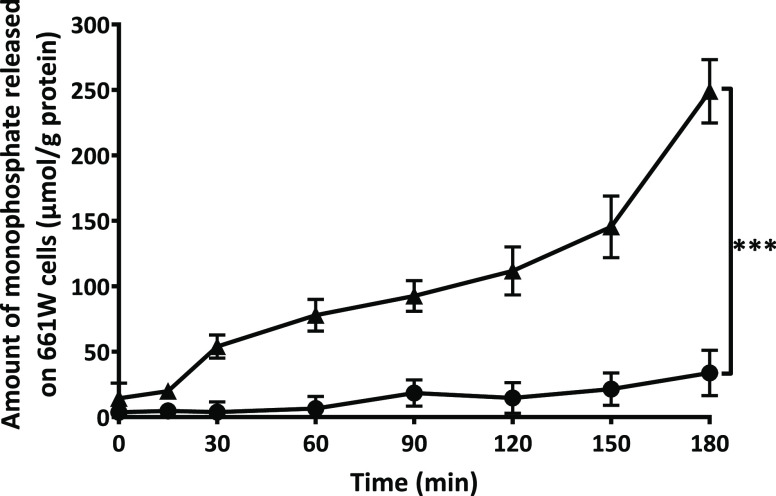
Release of monophosphates from TPP-coated PLOA-decorated NEs induced by membrane-bound enzymes in 661W cells (▲). Reduced monophosphate release in the presence of 0.5% v/v phosphatase inhibitor cocktail 2 (●). Data are expressed as means ± SD, *n* ≥ 3. The significant difference after 3 h is indicated as ***p < 0.001.

We further confirmed that monophosphates were also released from the surface of PLOA/TPP NEs in cell culture. Using 661W cells as a model system, the monophosphate release into the cell culture medium increased steadily over time without reaching a plateau after 3 h (Figure 9). Membrane-bound enzymes were efficiently inhibited, showing a 7.4-fold lower monophosphate release after 3 h. However, complete inhibition of enzyme activity was not attained, a finding that is in agreement with the results from previous research on Caco-2 and HEK-293 cell lines.^36,37^

### 3.5 Cell Viability Assay

**Figure 10:**
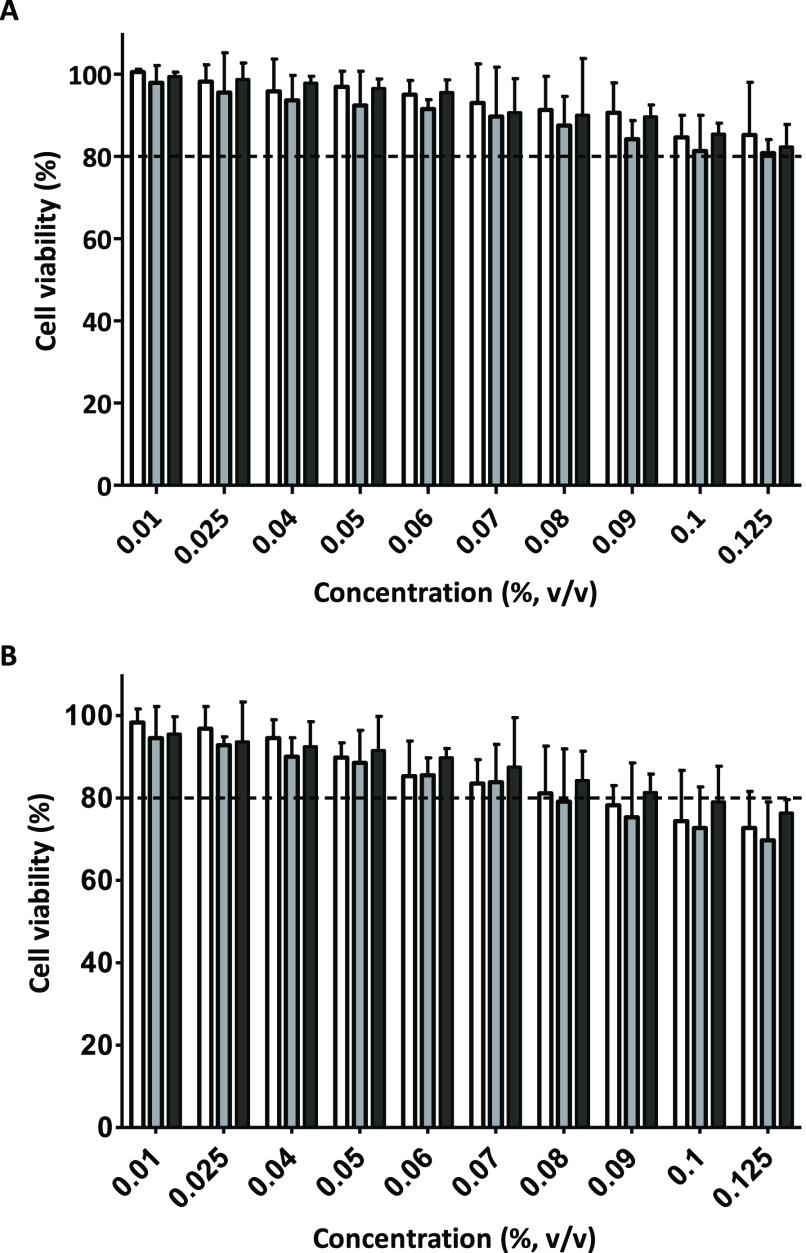
Concentration and incubation time-dependent cytotoxicity of NEs on 661W cells. (A) Cells were treated with NEs for 1 h. (B) Cells were treated with NEs for 3 h. White bars: control NEs, gray bars: PLOA-decorated NEs, and black bars: TPP-coated PLOA-decorated NEs. Data are expressed as mean ± SD, *n* ≥ 3.

As a next step, we tested the cell viability of 661W cells by incubating them for 1 h and 3 h with 10 NE concentrations in the range of 0.01–0.125% v/v (Figure 10). After 1 h of incubation, cell viability was above 90% for all tested concentrations and without significant difference in cell viability between the three NE formulations (*p* < 0.05, Student’s *t*-test). These data suggested that 1 h of exposure to NEs is safe for further experiments. At NE concentrations ≤0.06% v/v, cell viabilities are higher than 90% after 1 h and 3 h. At a NE concentration ≥0.08%, cell viabilities after 3 h fell below 80%, indicating potential cytotoxicity.

Cationic CPPs in general were considered to be toxic to most types of cells.^38,39^ The concentration and structure of CPPs together with nanocarrier properties are factors that affect their toxicity. Polyamine groups on the CPP backbone can bind to glycosaminoglycan substructures on the cell membrane, resulting in membrane perturbation and thus inducing cell toxicity.^40^ Recent findings, however, showed that not only conjunctival and corneal cells but also endothelial and retinal cells tolerated CPPs quite well.^38,40,41^ This observation may explain the similar cytotoxic properties of (i) PLOA NEs where PLL substructures are expressed on the NE surface, (ii) PLOA/TPP NEs with PLL substructures being covered by TPP, and (iii) control NEs loaded with fatty amine OA. Besides, loading the PLL conjugate into NEs may also reduce the toxicity of the PLL substructures. Le-Vinh et al. showed that immobilization of a phosphorylated PEG surfactant on the solid lipid nanoparticle surface causes a much lower cytotoxic effect on cells compared to that of the same surfactant in a free state.^36^

### 3.6 Cellular Uptake Studies of CPP-Decorated NEs

**Figure 11:**
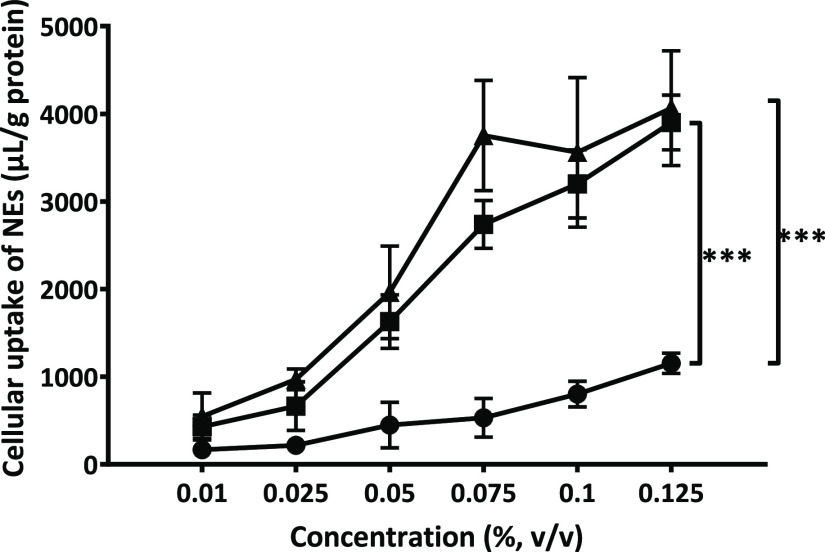
Cellular uptake of NEs into 661W cells at different concentrations by fluorescence spectroscopy. Cells were incubated with PLOA-decorated NEs (▲), TPP-coated PLOA-decorated NEs (■), and control NEs (●) for 1 h. Data are expressed as means ± SD, *n* ≥ 3. The significant difference is indicated as ***p < 0.001.

As obvious from the cellular uptake profile, NE decoration with PLOA significantly increased their internalization (Figure 11). The positively charged PLOA NEs showed the highest level of NE internalization of all, wherein cellular uptake levels were already saturated at 0.075% v/v NEs because we found no significant difference between 0.075, 0.1, and 0.125% v/v NEs (*p* > 0.05, Student’s *t*-test). The negatively charged PLOA/TPP NEs showed comparable cellular uptake profiles as PLOA NEs, although it seemed that saturation was not yet reached at a concentration of 0.125% v/v. Along with monophosphate cleavage and surface-exposed amine results, these findings provide further evidence that we uncovered PLL on NE surfaces.

**Figure 12:**
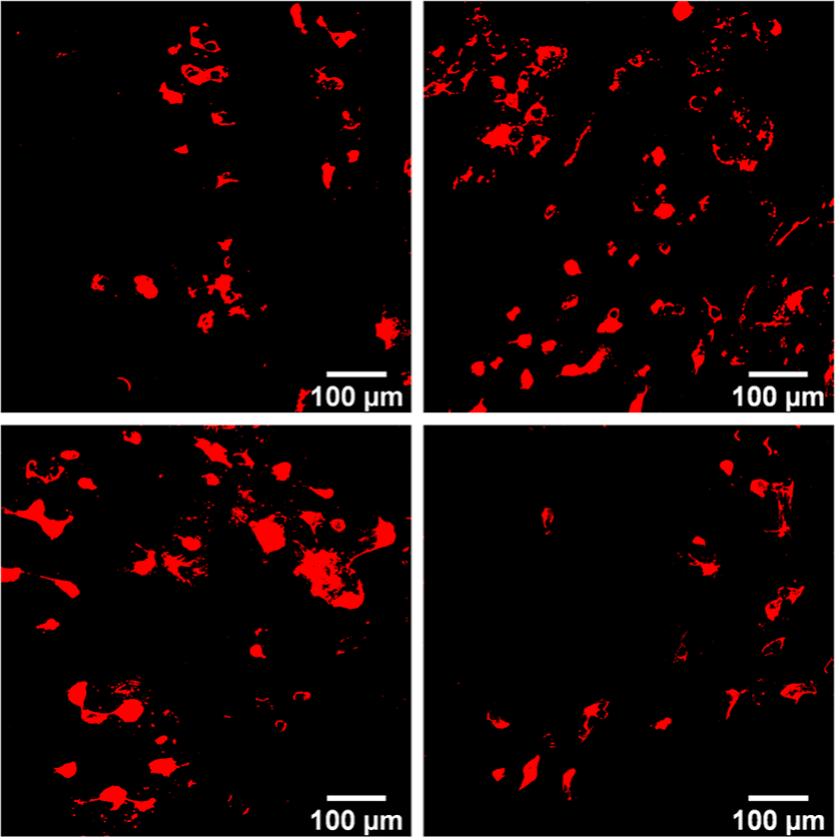
Microscopic imaging of 661W cells incubated with Lumogen red-loaded NEs diluted to 0.005% v/v: control NEs (A), PLOA-decorated NEs (B), TPP-coated PLOA-decorated NEs (C), and TPP-coated PLOA-decorated NEs +0.5% v/v phosphatase inhibitors cocktail 2 (D).

Microscopic imaging was utilized to visualize the cellular uptake efficiency of control NEs, PLOA NEs, and PLOA/TPP NEs at a concentration of 0.005% v/v. Red fluorescent signals from PLOA NE- and PLOA/TPP NE-treated cells indicated a stronger accumulation compared to control NEs (Figure 12A vs. B,C). Conversely, the fluorescence signal decreased when 661W cells were coincubated with 0.5% v/v PIC2 (Figure 12D).

**Figure 13:**
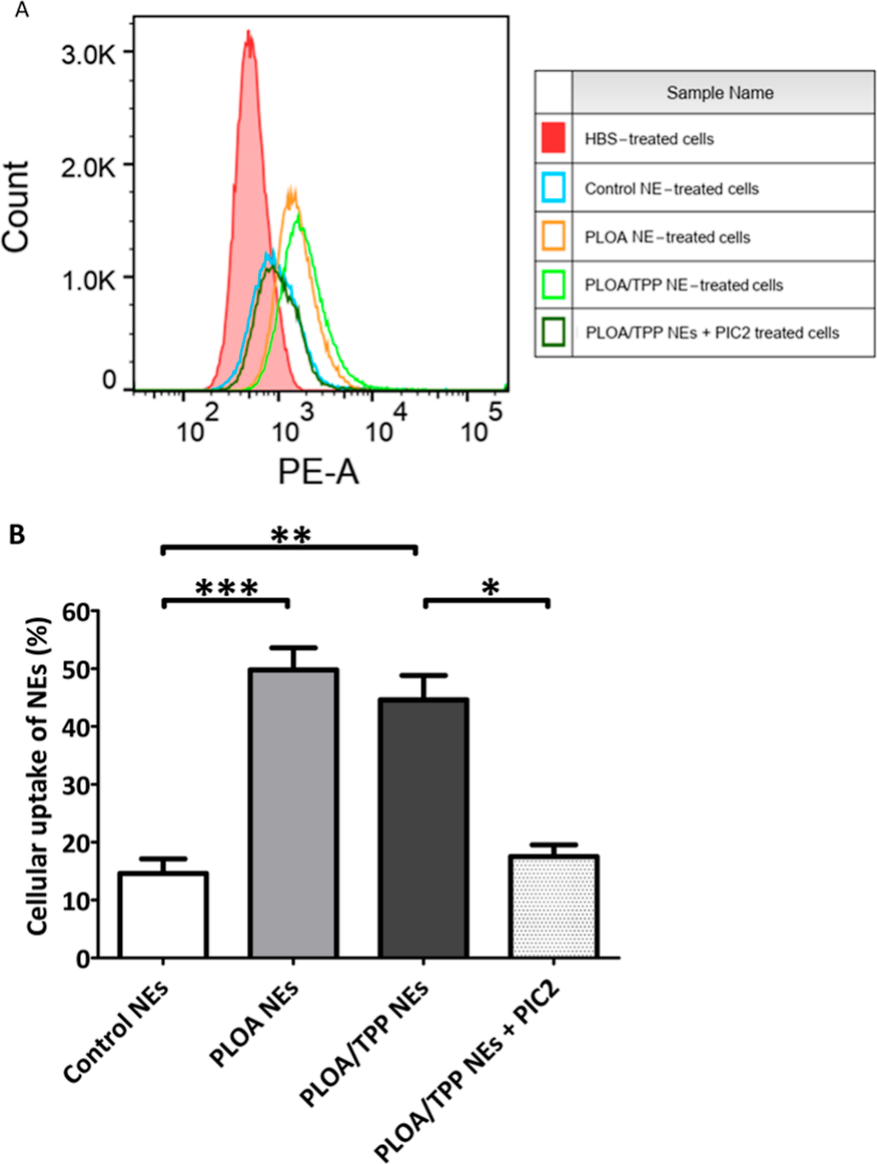
Cellular uptake of Lumogen red-loaded NEs by flow cytometry. (A) Single parameter histograms of PLOA-decorated NEs (PLOA NEs, orange), TPP-coated PLOA-decorated NEs (PLOA/TPP NEs, light green), PLOA/TPP NEs +0.5% v/v phosphatase inhibitor cocktail 2 (PIC2) (dark green), control NEs (blue), and HBS (red). Count stands for the number of events. A phycoerythrin-area channel (PE-A) was used as a detection parameter for Lumogen red-loaded NE-positive cells. (B) Percentage of cells that showed uptake of NEs at a 0.05% v/v dilution. Data are expressed as means ± standard deviations, *n* ≥ 3. The significant differences are indicated as *p < 0.05, **p < 0.01, ***p < 0.001.

Furthermore, the cellular uptake efficiency was assessed by flow cytometry using a defined gating strategy, illustrated in Figure S.1, to identify events corresponding to the fluorescent molecule uptake of single photoreceptor-like cells. A notable shift of the fluorescence signal is seen in Figure 13. A indicates an increase in fluorescence intensity when cells were incubated with PLOA NEs and PLOA/TPP NEs as compared to control NEs. Figure 13. B illustrates the cellular uptake profile of all formulations at the concentration of 0.05% v/v. The results showed that PLOA NEs were internalized into ∼50% of the 661W cell population, which is 3.5-fold higher than that of control NEs, whereas PLOA/TPP NEs were taken up by ∼45% of the cell population. The addition of 0.5% v/v PIC2 to samples incubated with PLOA/TPP NEs led to a remarkable decrease in uptake efficiency into 661W cells with only 17% of the cell population taking up NEs.

### 3.7 Gene Transfection Study

**Figure 14:**
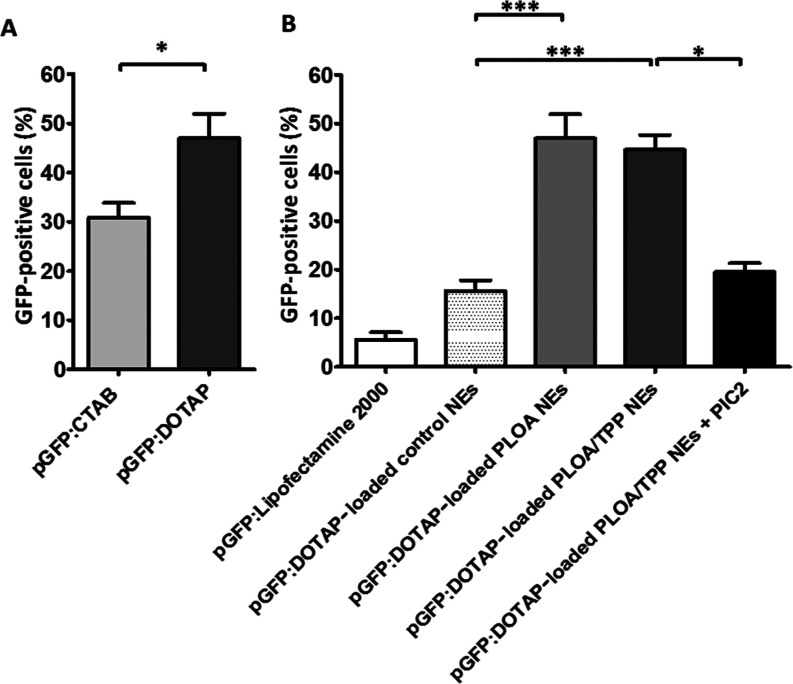
(A) Comparison of transfection efficiencies between pGFP/DOTAP- and pGFP/CTAB-loaded PLOA-decorated NEs. (B) Transfection efficiencies of pGFP/DOTAP-loaded different NEs after 1 h incubation on 661W cells as analyzed by flow cytometry. The amount of pGFP in each well was kept at ∼200 ng. Lipofectamine 2000 was used as a positive control. Control NEs, PLOA-decorated NEs (PLOA NEs), and TPP-coated PLOA-decorated NEs (PLOA/TPP NEs) were diluted to the same concentration of 0.05% v/v. The presence of CPP in PLOA NEs led to ∼2.5 times higher transfection efficacy than control NEs. Coating PLOA NEs with TPP did not show a significant change in the number of transfected cells. The copresence of phosphatase inhibitor cocktail 2 (PIC2) at 0.5% with PLOA/TPP NEs led to ∼50% lower in transfected cells, confirming the role of ALP as a partner for gene transfection of PLOA/TPP NEs. Data are expressed as means ± standard deviations, *n* ≥ 3. The significant differences are indicated as *p < 0.05, ***p < 0.001.

In order to load pGFP into the lipophilic phase of NEs, DOTAP or CTAB was used to form hydrophobic ion pairs with pGFP to increase the lipophilicity of pGFP. As illustrated in Figure 14A, pGFP/DOTAP showed a significantly higher gene transfection efficacy than pGFP/CTAB (*p* < 0.05, Student’s *t*-test). This result is in agreement with the already published literature, showing that DOTAP can yield higher gene transfection levels than CTAB because of the two fatty chains in the DOTAP structure, while CTAB is a single-chain cationic surfactant.^42−44^ Therefore, the pGFP/DOTAP complex was chosen for further transfection experiments to test gene transfection efficiencies of pGFP/DOTAP-loaded NEs diluted at 0.05% v/v. Comparable to our cellular uptake experiments with Lumogen red (Figure 13B), we found that PLOA NEs showed the highest transfection efficiency followed by PLOA/TPP NEs and then control NEs (Figure 14B). In the presence of 0.5% v/v PIC2, the transfection efficiency of PLOA/TPP NEs significantly decreased to the same range as that of the control NEs. The positive control pGFP/Lipofectamine 2000 yielded an even lower transfection efficacy than the control NEs (*p* < 0.01, Student’s *t*-test). This can be explained by (i) the suboptimal Lipofectamine 2000 to pGFP ratio, (ii) the cell-type dependent effect, and/or (iii) the short transfection time of 1 h. As an amount of more than 0.25 μL of Lipofectamine 2000 per well showed signs of cytotoxicity to 661W cells, the Lipofectamine used in this experiment was 0.125 μL/well, meaning that the pGFP/Lipofectamine 2000 ratio was 1:0.6 that was lower than the commonly used 1:2 or 1:3 ratio. Cytotoxicity of Lipofectamine 2000 has been widely acknowledged, especially when low cell density was employed.^45−47^ Another factor that was also reported is the considerable difference between Lipofectamine 2000 batches both in toxicity and also transfection efficiency.^48^

## 4 Conclusions

ALP-responsive charge-converting NEs were for the first time applied to deliver drug and plasmid DNA to retinal cells, showing promising results in drug delivery and gene transfection efficacy. In this study, a lipophilic modification of hydrophilic PLL at its carboxylic end with oleylamine was performed, enabling the loading of PLOA molecules into the lipophilic preconcentrate and the expression of PLL moieties on the surfaces of NE droplets. The functional polyphosphate coating layer on PLOA/TPP NEs covering the positively charged PLL can help the nanocarriers to traverse the vitreous humor with less risk of interacting with negatively charged components in the vitreous humor and facilitating the access of cell membrane-bound ALP, leading to the almost complete reversion of ζ potential or to be precise, the almost complete recovery of CPP on the NE surface. As a result, cellular uptake of NEs was significantly enhanced in photoreceptor-like cells. Moreover, the hydrophobic ion pair pGFP–DOTAP complexes were prepared and loaded into PLOA/TPP NEs, resulting in high GFP expression with ∼50% of cells transfected. Furthermore, with their low cytotoxicity, ALP-responsive charge-converting NEs could be a potential functional carrier for retinal drug and gene delivery.

## Supporting Information Available

Gating strategy for flow cytometry based on the area of forward versus side scatter (FSC-A vs SSC-A) and cellular uptake profile of Lumogen red-loaded NEs with and without polyphosphate coatings compared to control NE in the presence and absence of PIC2 (PDF)
