# Supplementary material for: Charge-Converting Nanoemulsions as Promising Retinal Drug and Gene Delivery Systems
Source: ACS Appl Mater Interfaces. 2022 Sep 20;14(39):44981–91. doi: 10.1021/acsami.2c11649 (PMC9542710; doi:10.1021/acsami.2c11649)
Supplement: Supplementary file 1 — am2c11649_si_001.pdf [file am2c11649_si_001.pdf]

SUPPORTING INFORMATION

Charge-converting nanoemulsions as promising retinal drug and gene delivery systems

Nguyet-Minh Nguyen Le [a], Sarah Zsák [b], Bao Le-Vinh [a], Julian David Friedl [a], Gergely Kali [a], Patrick Knoll [a], Hartwig Wolfram Seitter [b], Alexandra Koschak [b,*] and Andreas Bernkop-Schnürch [a,*]

[a] Center for Chemistry and Biomedicine, Department of Pharmaceutical Technology, Institute of Pharmacy, University of Innsbruck, Innrain 80/82, 6020 Innsbruck, Austria

[b] Center for Chemistry and Biomedicine, Department of Pharmacology and Toxicology, Institute of Pharmacy, University of Innsbruck, Innrain 80/82, 6020 Innsbruck, Austria

[*] shared correspondence: Alexandra Koschak, University of Innsbruck, Institute of Pharmacy, Pharmacology and Toxicology, Center for Chemistry and Biomedicine, Innrain 80-82, 6020 Innsbruck Austria, phone: +43-(0)512-507-58807, fax: +43-(0)512-507-58899, email: alexandra.koschak@uibk.ac.at and Andreas Bernkop-Schnürch, University of Innsbruck, Institute of Pharmacy, Pharmaceutical Technology, Center for Chemistry and Biomedicine, Innrain 80-82, 6020 Innsbruck Austria, phone: +43-(0)512-507-58601, fax: +43-(0)512-507-58699, email: andreas.bernkop@uibk.ac.at

Gating strategy for flow cytometry

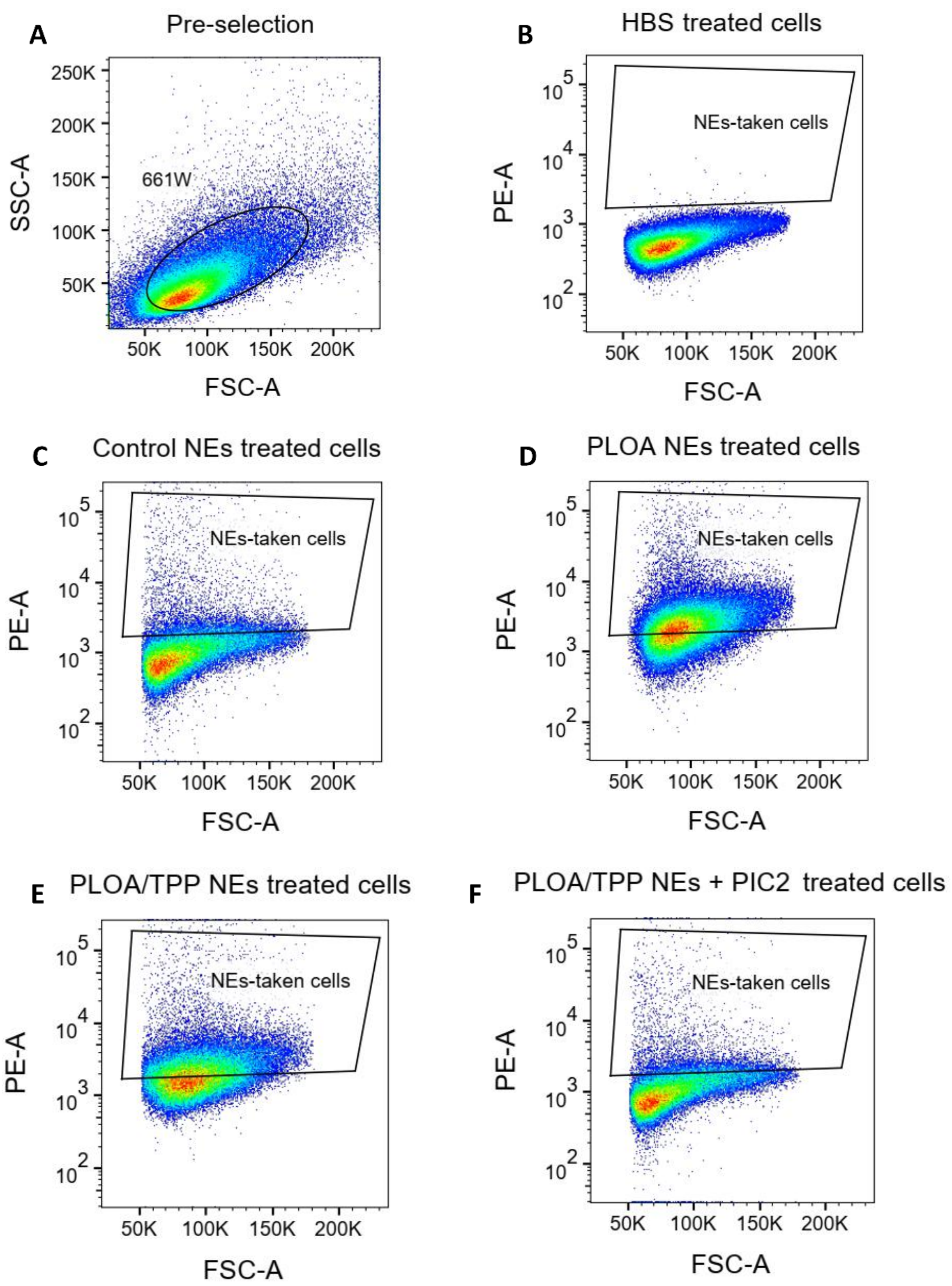

Figure S.1. Gating strategy for flow cytometry. Cellular uptake profile of Lumogen red loaded NEs. FSC-A: forward scatter-area. SSC-A: side scatter-area. PE-A: phycoerythrin-area channel

used as detection parameter for Lumogen red loaded NEs-positive cells. (A) Pre-selection of main cell populations based on FSC-A and SSC-A followed by doublet-elimination based on FSC-Width (not shown). (B) HBS-treated 661W cells served as control and for setting the gate for fluorescence-positive cells. (C) Control NEs-taken 661W cells show only a small percentage of fluorescence-positive cells within the gate. (D) PLOA NEs-taken 661W cells and (E) PLOA/TPP NEs-taken 661W cells show substantial fractions of fluorescence-positive cells within the gate. (F) PLOA/TPP NEs-taken 661W cells in samples incubated with phosphatase inhibitor cocktail 2 show a small number of fluorescence-positive cells within the gate.

Calculation for transfection study on 661W photoreceptor-like cells

Each well was incubated with 250 µL of either 0.05% nanoemulsion, pDNA:Lipofectamine® 2000 liposome solution, or pDNA solution.

Below is the calculation of the amount of pDNA in each 250 µL solution of each type:

1. pGFP:DOTAP loaded NE formulation

Molar ratio DOTAP:pGFP ion pair = 2

MW (molecular weight): pGFP = 6100

DOTAP = 698.56

→ DOTAP-pGFP ion-pair ~ 7497.12

Concentration of loaded DOTAP-pGFP in SEDDS preconcentrate =	0.2	%
Amount of pGFP in each 100 mg lipophilic preconcentrate =	0.163	mg
1 µL lipophilic preconcentrate weighs =	0.97	mg

Each well incubated with 250 µL of 0.05% nanoemulsion

This amount of NE contains =	0.125	µL preconcentrate
→ equivalent to:	0.121	mg preconcentrate
→ equivalent to:	196.3	ng pGFP

2. pGFP:Lipofectamine® 2000 formulation

Concentration of Lipofectamine® 2000 reagent =	1	µg/µL
Concentration of pGFP solution =	4.30	µg/µL
0.3 µL of pGFP solution contains =	1290	ng pGFP

Mix 0.75 µL of Lipofectamine® 2000 stock solution and 0.3 µL of pGFP solution in 100 µL of buffer. Incubate for 5 min. Dilute pGFP:Lipofectamine® 2000 complex with buffer to the final volume of 1500 µL.

Final Lipofectamine® 2000 concentration =	0.05	%

250 µL pGFP:Lipofectamine® 2000 solution contains =	215	ng pGFP

3. pGFP solution alone

Concentration of pGFP solution =	4.30	µg/µL
0.3 µL of 4.3 µg/µL pGFP solution contains =	1.29	µg pGFP
→ equivalent to =	1290	ng pGFP

Mix 0.3 µL of pGFP solution with buffer to the final volume of 1500 µL.

→ 250 µL of pGFP diluted solution contains =	215	ng pGFP
